# The potential of metabolic engineering for sustainable phytosterol production

**DOI:** 10.1007/s44307-026-00108-9

**Published:** 2026-04-16

**Authors:** Runmeng Hu, Xueni Di, Guangli Li, Hubert Schaller, Andréa Hemmerlin, Pan Liao

**Affiliations:** 1 Department of Biology, Hong Kong Baptist University, Kowloon Tong, Hong Kong SAR, China; 2 Institut de Biologie Moléculaire Des Plantes (IBMP), Centre National de La Recherche Scientifique, Université de Strasbourg, 12 Rue du Général Zimmer, 67084 Strasbourg, France; 3 State Key Laboratory of Agrobiotechnology (CUHK), Hong Kong SAR, China; 4 AoE Centre for Plant Vacuole Biology and Biotechnology, The Chinese University of Hong Kong, Shatin, Hong Kong SAR, China; 5 Institute of Systems Medicine and Health Sciences, Hong Kong Baptist University, Kowloon Tong, Hong Kong SAR, China

**Keywords:** Phytosterol, Biosynthesis, Metabolic Engineering, Heterologous Production

## Abstract

Phytosterols, essential components pivotal to plant membrane stability and celebrated for their extensive pharmacological benefits, have garnered considerable attention across industries, including food fortification, nutraceuticals, and pharmaceuticals. The escalating demand for phytosterols, fueled by their myriad health advantages, underscores the urgent need for more efficient synthesis methodologies. Among these, metabolic engineering stands out as a promising approach due to its biologically driven process, which operates under stable conditions, thereby enhancing reaction specificity and drastically reducing the production of undesirable by-products. This review consolidates the latest research endeavors focused on enhancing phytosterol accumulation, providing a comprehensive analysis of strategies including gene manipulation, enzyme engineering, metabolic engineering, and the utilization of diverse host organisms such as bacteria, algae, and yeast. We explore recent advancements in phytosterol biosynthesis and engineering, providing a comprehensive overview of the field’s current state and examining promising methodologies for future research and applications.

## Introduction

Phytosterols are a group of naturally occurring steroids found throughout plant tissues, including roots, stems, leaves, flowers, fruits, and so on. These compounds predominantly occupy the membrane structures as free sterols, sterol esters, steryl glycosides, and acylated steryl glycosides, contributing to the structural and functional stability of plant membranes (Yang et al. 2018). The most prevalent phytosterols are β-sitosterol, campesterol, and stigmasterol, with β-sitosterol being the most abundant in plants (Miras-Moreno et al. 2016; Wang et al. 2018). In addition to their presence in plants, phytosterols have been identified in certain lower animals, such as snails or worms, suggesting a broader biological distribution (Jarusiewicz et al. 2005; Michellod et al. 2023). Recently, Michellod et al.’s research has challenged this assumption by demonstrating that certain marine annelids, specifically Olavius and Inanidrilus, can synthesize the phytosterol sitosterol de novo (Michellod et al. 2023). The presence of cholesterol in various organisms is well-documented, with the compound primarily biosynthesized in animals. Notably, cholesterol has been documented in several plant genera, including Solanum (Sonawane et al. 2016), Dioscorea (Johnson et al. 1963), Ypsilandra, Polygonatum (Yang et al. 2019), and Suaeda (Al-Mohammadi 2006), though its occurrence and functional role in plants remain less studied compared to phytosterols. Ergosterol is a sterol found predominantly in fungi, including edible mushrooms (Rangsinth et al. 2023), but it can also be produced by Chlamydomonas in response to endoplasmic reticulum (ER) stress (Je et al. 2024).

Structurally, phytosterols are triterpenes characterized by a perhydrocyclopentanophenanthrene ring system. This system features a hydroxyl group at the C-3 position, a side chain of 8–10 carbon atoms at the C-17 position, and typically a double bond at the C-5 position (Mohammadi et al. 2020). Phytosterols share a close resemblance to cholesterol. However, distinct structural differences set them apart: phytosterols typically possess an additional methyl or ethyl group at the C-24 position, and certain prevalent phytosterols, such as sitosterol and stigmasterol, exhibit an additional double bond at the C-22 position. These variations contribute to the unique biological functions and properties of phytosterols compared to cholesterol (Comunian and Favaro-Trindade 2016). To date, the phytosterol compendium has expanded to encompass over 250 distinct molecular entities, each with unique variations in their carbon side chains and the presence or absence of double bonds (Sharma et al. 2021; Tan and Men 2026). These compounds are broadly sorted into two primary categories: sterols, which are unsaturated due to the presence of double bonds, and stanols, which are fully saturated without any double bonds (Moreau et al. 2002). Further classification of phytosterols is based on the number of methyl groups at C-4 position, yielding the following subcategories: 4-desmethyl sterols, which are devoid of a methyl group at C-4 position, and include compounds such as β-sitosterol, stigmasterol, campesterol, and brassicasterol. While 4α-monomethyl sterols, which feature a singular methyl group at the C-4 position, with examples being gramisterol, lophenol, and citrostadienol. 4,4-dimethyl sterols, which have two methyl groups at C-4 position, and include molecules like cycloartanol, cycloartenol, and 2,4-methylenecycloartanol (Svoboda et al. 1995).

The extensive pharmacological properties of phytosterols have made them essential components in dietary supplements, nutritionally enriched food products, and cosmetic formulations. Recognized for their crucial role in enhancing cardiovascular health through the reduction of Low-Density Lipoprotein Cholesterol (LDL-C) and total cholesterol levels, phytosterols exhibit the capacity to hinder cholesterol absorption, which subsequently decreases, diminishing the risk of coronary heart disease. Notably, research indicates that the daily intake of 2 g of phytosterols can effectively lower low-density lipoprotein cholesterol levels by approximately 7 to 10% (Jones et al. 2000, National Cholesterol Education Program Expert Panel 2002, Cedó et al. 2019, Poli et al. 2021, Xia et al. 2022, Nakano et al. 2025, Rahman et al. 2025). Beyond their cardiovascular benefits, phytosterols exhibit anti-inflammatory effects, offering potential treatments for diseases such as rheumatoid arthritis and asthma (Ghaedi et al. 2020; Marahatha et al. 2021). Phytosterols and other steroidal compounds can regulate glycolipid metabolism, inflammation, and oxidative stress, thereby improving insulin resistance and lowering blood pressure (Nattagh‐Eshtivani et al. 2022; Jin et al. 2025). Their anticancer potential is also notable, with studies showing their ability to diminish tumor growth and the spread of metastasis in breast and colorectal cancers (Shahzad et al. 2017; Cioccoloni et al. 2022). Additionally, phytosterols may play a role in neuroprotection (Fakhri et al. 2021; Sharifi-Rad et al. 2022). The possibility that they could impede the aggregation of proteins implicated in Alzheimer's disease adds another layer to their therapeutic promise (Uddin et al. 2020; Sharifi-Rad et al. 2022). Over the past decade, the functional food industry has increasingly incorporated phytosterols into a variety of daily food products, including yogurts, cereals, spreads, salad dressings, and many breakfast items (Altaf et al. 2025). A wide array of phytosterol-fortified foods, including dairy products, spreads, and baked goods, have since found success in over twenty countries, backed by endorsements from regulatory bodies like the FDA and the European Commission for their cardiovascular benefits (García-Llatas and Rodríguez-Estrada 2011; Duong et al. 2016; Ghaedi et al. 2020). The global phytosterols market was assessed to be worth more than $700 million in 2019 and is expected to experience a compound annual growth rate (CAGR) of 8.7% from 2020 to 2027 (https://www.grandviewresearch.com/industry-analysis/phytosterols-market). Optimizing the production of phytosterols not only responds to this demand but also holds the potential to deliver valuable insights for industrial processes.

The synthesis of phytosterol esters is predominantly conducted through chemical (Yang et al. 2016; Liu et al. 2021; Zhi and Wu 2021) and biological methods (Miras-Moreno et al. 2016; Pereira et al. 2022; Xu et al. 2022). Traditional solvent extraction methods, such as the Soxhlet method and cold pressing, commonly suffer from issues including high solvent consumption, significant energy expenditure, lengthy operational cycles, and environmental pollution (Uddin et al. 2018). Additionally, due to the low natural content of phytosterols in raw materials, large quantities of raw materials are required to achieve higher yields, while production is also susceptible to fluctuations in raw material sources and seasonal variations (Xinyue 2024). Although chemical synthesis allows for structural modification, it often involves complicated processes, frequent side reactions, and low selectivity. Additionally,due to the non-specific nature of chemical catalysts, such conditions can lead to the formation of undesirable by-products, including dienes, trienes, or phytosterol oxides (Meng et al. 2006; He et al. 2014; Pereira et al. 2022). These by-products not only reduce the overall yield but also add complexity to the purification processes, presenting a significant drawback to this method (Wang and Lu 2018). Modern green extraction technologies, such as supercritical fluid extraction and enzyme-assisted extraction, have improved certain issues, but they still struggle to fundamentally resolve raw material constraints and environmental burdens (Uddin et al. 2018). Through gene editing (e.g., CRISPR), directed enzyme evolution, and microbial cell factory construction, precise regulation of key enzymes and pathways has been achieved, significantly enhancing target product yield and purity.

Recent advancements have highlighted biosynthesis as a method of particular interest due to its selectivity and reduced environmental impact. This biologically driven process is characterized by its operation under comparatively mild conditions, which not only enhances the specificity of the reactions but also significantly reduces the generation of unwanted by-products. Notably, the application of various lipases in the enzymatic synthesis of phytosterol esters has been shown to be effective, marking this approach as a more ecologically sustainable and potentially safer route to produce these compounds. Through this review, we aim to delineate the progress made in biosynthetic methods, emphasizing the implications and potential for future developments in the synthesis of phytosterols. We present a comprehensive overview of the current state of research in the field of phytosterol biosynthesis.

## Biosynthesis of Phytosterols

### Overview of Phytosterol Biosynthesis

**Fig. 1:**
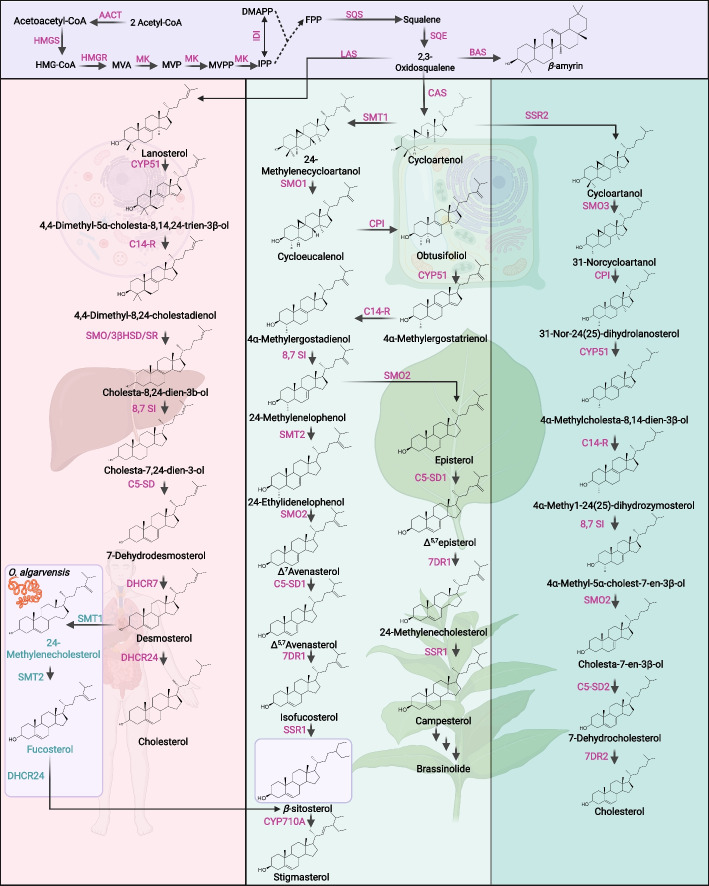
The phytosterol and cholesterol biosynthetic pathway and some phytosterol derivatives in plants. The diagram depicts the metabolic flow from Acetyl-CoA through the MVA pathway to 2,3-oxidosqualene (represented by the purple regions). This pathway leads into the cholesterol biosynthesis pathway in animals (shown in pink), the phytosterol biosynthesis pathway (in green), and the cholesterol biosynthesis pathway in plants (in yellow). Dotted lines indicate multi-step synthesis processes, whereas solid lines represent single-step synthesis processes. Biosynthetic genes are marked in deep pink, highlighting their role in catalytic steps. Metabolites and intermediates are labeled in black. 3βHSD: 3β-hydroxysteroid dehydrogenase; 7DR1: Δ^7^-sterol reductase 1; 7DR2: Δ^7^-sterol reductase 2; 8,7 SI: Sterol Δ^8^-Δ^7^ isomerase; AACT: Acetyl-CoA acetyltransferase; BAS: β-amyrin synthase; C14-R: Sterol C-14 reductase; C5-SD1: Sterol C-5 desaturase 1; C5-SD2: Sterol C-5 desaturase 2; CAS: Cycloartenol synthase; CPI: Cycloeucalenol isomerase; CYP51: Sterol 14α-demethylase; CYP710A: Sterol C-22 desaturase; DHCR7: 7-dehydrocholesterol reductase; DHCR24: desmosterol reductase; DMAPP: Dimethylallyl diphosphate; FPP: Farnesyl diphosphate; HMGR: 3-hydroxy-3-methylglutaryl-CoA reductase; HMGS: 3-hydroxy-3-methylglutaryl-CoA synthase; IDI: Isopentenyl diphosphate isomerase; IPP: Isopentenyl diphosphate; LAS: Lanosterol synthase; MK: Mevalonate kinase; MVA: Mevalonate; MVP: Mevalonate 5-phosphate; MVPP: Mevalonate 5-diphosphate; SMO: Sterol methyl oxidase; SMO1: Sterol methyl oxidase 1; SMO2: Sterol methyl oxidase 2; SMO3: Sterol methyl oxidase 3; SMT1: Sterol methyltransferase 1; SMT2: Sterol methyltransferase 2; SQE: Squalene epoxidase; SQS: Squalene synthase; SR: Sterol reductase; SSR: Sterol side-chain reductase. Created in https://BioRender.com

The biosynthesis of phytosterols in plants entails a sophisticated series of enzyme-catalyzed reactions. Phytosterols are primarily synthesized within the ER, predominantly through the cycloartenol pathway, which unfolds in three distinct enzymatic stages (Valitova et al. 2016). The biosynthesis of phytosterols could mainly be seen in Fig. 1. Universally, the biosynthesis of sterols is mainly derived from the mevalonate (Kittipongpittaya, et al., 2025) pathway, wherein two molecules of isopentenyl diphosphate (IPP) are transferred to its allylic isomer, dimethylallyl diphosphate (DMAPP), to form farnesyl diphosphate (FPP) (Hemmerlin et al. 2012; Zhao et al. 2013). In higher plants and algae, sterol biosynthesis is enlarged by an alternative route: IPP and DMAPP may originate not only from the cytosolic MVA pathway but also from the 2-*C*-methyl-d-erythritol 4-phosphate (MEP) pathway within the plastids (Rohmer 1999; Hemmerlin et al. 2012). Intriguingly, diatoms demonstrate variability in this biosynthetic divergence; certain species can utilize both the MVA and MEP pathways, whereas others are restricted to sterol synthesis via the MVA pathway. This metabolic versatility underscores the evolutionary adaptations of sterol biosynthesis across different plant and algal taxa (Vranová et al. 2013; Zhao et al. 2013; Gallo et al. 2020).

The MVA pathway begins with the condensation of acetyl-CoA units to form acetoacetyl-CoA, which is subsequently transformed into 3-hydroxy-3-methyl glutaryl-CoA (HMG-CoA). The rate-limiting step, governed by HMG-CoA reductase (HMGR), converts HMG-CoA into mevalonic acid (Kittipongpittaya et al*.* 2025). Specific inhibitors of HMGR, such as statins, have been widely used as cholesterol-lowering medicine till now (Friesen and Rodwell 2004). Through several phosphorylation and decarboxylation reactions, mevalonate is converted into IPP, the universal precursor to various isoprenoids. IPP is then isomerized to DMAPP, setting the stage for subsequent synthesis of more complex terpenes and sterols (Learned and Fink 1989; Rohmer 1999). MEP pathway initiates with the rate-limiting enzyme 1-deoxy-d-xylulose 5-phosphate synthase (DXS), which catalyzes the condensation of pyruvate and glyceraldehyde-3-phosphate into 1-deoxy-d-xylulose 5-phosphate (DXP) (Cordoba et al. 2009). Following this, DXP is converted into 2-*C*-methyl-d-erythritol 2,4-cyclodiphosphate (MEcPP) through a series of enzyme-mediated reactions that include isomerization, phosphorylation, and cyclization (Takahashi et al. 1998). The final products of the pathway, IPP and DMAPP, are generated from ME after several reductions and dephosphorylation steps. A series of head-to-tail condensations occurs, involving multiple molecules of IPP with DMAPP, to form the 15-carbon intermediate FPP under the action of farnesyl diphosphate synthase (FPPS) (Eisenreich et al. 2004). Two FPP molecules are then joined in a head-to-head condensation by the enzyme squalene synthase (SQS), resulting in the formation of squalene, a 30-carbon linear triterpene (Busquets et al. 2008). Although FPPS and SQS are generally not considered as rate-limiting as HMG-CoA reductase and DXS, these enzymes still play important roles and can be regulated (Lu et al. 2020). The following step is the cyclization of squalene via squalene epoxidase (SQE) to form 2,3-oxidosqualene (Phillips et al. 2006). Cycloartenol synthase (CAS), a category of oxidosqualene cyclase (OSC) (Pisoschi 2024) converts 2,3-oxidosqualene to cycloartenol, the initial sterol precursor in plants (Corey et al. 1993). Cycloartenol is modified through an analogous series of enzymatic steps, leading to the formation of phytosterols such as β-sitosterol, campesterol, and stigmasterol. β-sitosterol synthesis primarily involves the enzyme sterol C-24 methyltransferase (SMT), which catalyzes the transfer of a methyl group to create a C28 sterol, followed by a series of reductions and demethylations (Bouvier‐Navé et al. 1997; Schaeffer et al. 2001). However, in recent studies, researchers have discovered the biosynthetic pathway of phytosterols in two non-intestinal animals, Olavius and Inanidrilus (Michellod et al. 2023). They identified a non-classical C24-SMT enzyme in these two marine invertebrates, which have not been previously found in bilaterally symmetrical animals. This suggests that the strict barrier between plants and animals for synthesizing phytosterols has been broken down. Interestingly, phylogenetic analysis of the C24-SMT homologs revealed the potential presence of similar enzymes in Cnidaria, Porifera, Rotifera, and Mollusca, suggesting that the biosynthesis of phytosterols is not exclusive to higher plants (Michellod et al. 2023). However, the physiological functions of auto-synthetic phytosterols in animals remain unexplored, which needs further research (Michellod et al. 2023). Campesterol is formed through a pathway involving C24 methylation via the SMT1 enzyme to generate the C28 side chain. Stigmasterol is synthesized from sitosterol by the action of the enzyme C22-desaturase, which introduces an additional double bond at the 22nd carbon position of the sterol side chain (Klahre et al. 1998). Sterol methyl oxidase (SMO) is involved in the removal of methyl groups from the C-4α position of sterol intermediates, a necessary step in the elaboration of the sterol structure and function and controls the level of 4,4-dimethylsterol and 4α-methylsterol precursors in photosynthetic eukaryotes (Darnet and Schaller 2019). These enzymes are highly specific and play crucial roles in the diversification of phytosterols, which are vital membrane components and act as precursors for plant hormones. On the other hand, cholesterol production has been observed in plants such as tomato, potato, tobacco, and *Nicotiana benthamiana* (Sonawane et al. 2016), suggesting that cholesterol biosynthesis may be more widespread in the plant kingdom.

### Regulation of enzymes in phytosterol biosynthesis

The biosynthesis of phytosterols is a multi-step, energy-intensive metabolic pathway. Along this lengthy and complex branching pathway, enzyme activities at multiple critical junctions undergo stringent regulation. Among these, HMGR represents the first key control point regulating the overall flux of isoprenoid synthesis (Hemmerlin 2013). Subsequently, in the downstream sterol-specific branch, SQS and SQE mark irreversible commitment steps directing metabolic flux toward sterol synthesis (Wentzinger et al. 2002; Unland et al. 2018). OSC catalyzes the conversion of linear precursors into cyclic sterol skeletons, representing a critical divergence point for forming diverse triterpenoid compounds (Guo et al. 2022). Furthermore, enzymes such as SMT and SMO directly influence the types and proportions of final sterols produced (Schaeffer et al. 2001; Lu et al. 2025a, b). Thus, precise regulation of key enzymes like HMGR, SQS, SQE, OSC, SMT, and SMO constitutes a central strategy for plants to achieve sterol homeostasis. This chapter will delve into the intricate regulatory mechanisms governing these enzymes at multiple levels, transcriptional, post-transcriptional, translational, and post-translational, elucidating how plants respond to endogenous developmental signals and external environmental stimuli.

### HMGR

The reduction of HMG-CoA to mevalonic acid relies on the reducing power of nicotinamide adenine dinucleotide phosphate (NADPH). This step is the overall rate-limiting factor in the MVA pathway, in which HMGR catalyzes this reaction (Stermer et al. 1994). As the principal enzyme in the MVA pathway, HMGR controls the flux of metabolites towards the biosynthesis of sterols, including phytosterols, and other isoprenoids (Burg and Espenshade 2011; Hemmerlin 2013; Kim et al. 2013; Kumar et al. 2018). The expression and activity of HMGR are tightly regulated through transcriptional, translational, post-translational mecahnisms, which are modulated by plant hormones, sterols, and exogenous chemicals,ensuring lipid homeostasis and adaptative responses to environmental stimuli (Burg and Espenshade 2011; Leivar et al. 2011; Hemmerlin 2013; Robertlee et al. 2017; Liu et al. 2024).

*HMGR* transcription in plants is regulated by a combination of promoter elements sensitive to environmental and developmental cues, differential expression of gene family members, and specific responses to stress and pathogen challenges. The *HMGR* gene in *Salvia miltiorrhiza* contains a promoter with motifs recognized by transcription factors responsive to light, salicylic acid (SA), bacterial infection, and auxins. This suggests that *HMGR* expression is regulated during flowering, embryogenesis, organogenesis, and the circadian rhythm (Majewska et al. 2022). HY5-PIF physically interacts with the *AtHMGR2* promoter to show opposite transcriptional regulation of the sterol biosynthetic pathway in Arabidopsis (Michael et al. 2024). Lin et al. (2023a) identified two novel transcription factors, GoHSFA4a and GoNAC42, in cotton glandular secretory cells using single-cell RNA sequencing. These TF specifically bind to the HSF motif within 3-hydroxy-3-methylglutaryl-CoA synthase (*HMGS*) promoter and the NAC motif within *HMGR* promoter, respectively. *Euphorbia kansui* is a traditional Chinese medicinal herb rich in β-sitosterol. The transcription factor, EkbHLH144, could bind to the *EkHMGR* promoter, and overexpressing it in Arabidopsis enhances β-sitosterol biosynthesis (Wang et al. 2024a). In vascular plants, *HMGR* gene family usually has multiple members and contribute to the diversity and specialization of *HMGR* functions, such as *Hevea brasiliensis* (Chye et al. 1992), Arabidopsis (Enjuto et al. 1994), *Gossypium raimondii* (Liu et al. 2018), *Lithospermum erythrorhizon* (Wang et al. 2023b), *Populus trichocarpa* (Ma et al. 2023), *Santalum album* (Niu et al. 2025) and *Ziziphus jujuba var. spinosa* (Tang et al. 2025). This indicates a complex transcriptional regulation allowing for tissue-specific expression.

Many studies concentrate exclusively on gene expression when examining the functioning of enzymes or metabolic pathways. However, this approach may not accurately reflect what occurs in living organisms and can sometimes lead to incorrect conclusions. In addition to gene expression, it is crucial to consider enzyme activity to gain a comprehensive understanding of these processes. In plant biology, the HMGR extends beyond transcriptional mechanisms and is primarily governed by post-translational modifications (Hemmerlin 2013). HMGR is subject to feedback inhibition and degradation, which is crucial for maintaining sterol homeostasis. This involves complex regulatory mechanisms, including interactions with protein kinases and phosphatases (Leivar et al. 2011; Foresti et al. 2013; Robertlee et al. 2017). In Arabidopsis, HMGR post-translational modifications, such as phosphorylation at the Ser577 site by SnRK1 (Robertlee et al. 2017; Robertlee 2018), ubiquitination by E3 Ubiquitin Ligase (SUD1), which could regulate HMGR activity through an ER-associated degradation (ERAD)-dependent mechanism (González Doblas 2012, Doblas et al. 2013). The same mechanism has also been found in Arabidopsis High Sterol Ester 1 (AtHiSE1) protein, which significantly lowers the levels of HMGR1 and HMGR2 proteins without impacting the transcript levels of the genes that encode these proteins (Shimada et al. 2019). Protein Phosphatase 2 A (PP2A) acts as both a positive and negative regulator of HMGR. It modulates HMGR activity through its B″ regulatory subunits, B″α and B″β, which interact with HMGR isoforms. PP2A influences HMGR activity during normal development and stress conditions (Cai et al. 2024). While brassinosteroids are not directly mentioned as regulators of HMGR, the enzyme is modulated by PP2A, which is involved in brassinosteroid signaling (Antolín-Llovera et al. 2011).

Some other plant hormones are also involved in HMGR regulation by post-translational regulation, such as gibberellic acid (GA) (Manoharlal et al. 2022), methyl jasmonate (MeJA) (Maldonado-Mendoza et al. 1994), and abscisic acid (ABA) (Kochan et al. 2019; Yang et al. 2020). These mechanisms ensure that HMGR activity is finely tuned in response to both internal and external stimuli, highlighting its critical role in plant metabolism and adaptation. Furthermore, exogenous chemicals, mevinolin (Hemmerlin et al. 2003) and other statins (Brain et al. 2006) can effectively regulate HMGR activity as HMGR-specific inhibitors in plant cell studies. Although HMGR is recognized as the upstream enzyme in the phytosterol biosynthetic pathway, it is subject to regulation by the phytosterols themselves (Russell and Davidson 1982). This negative feedback mechanism likely serves to maintain a relatively stable concentration of terpenoids within the system. HMGR is a pivotal enzyme in the phytosterol biosynthetic pathway, acting as a gatekeeper for the flow of metabolites through the MVA pathway. Its regulation is complex and involves multiple layers of control to ensure proper synthesis of sterols and isoprenoids, which are vital for plant growth, development, and stress responses. FPP, GGPP, and the final product sterol may also exert regulatory effects on HMGR, primarily through protein degradation (at the post-translational level) and partial transcriptional regulation. Both FPP and GGPP can regulate HMGR protein stability by promoting its ubiquitination and ERAD. Notably, GGPP has been demonstrated to be a potent endogenous HMGR degradation signal molecule in both yeast (Garza et al. 2009; Lu et al. 2025b) and mammalian systems (Jo and DeBose-Boyd 2022). In plants, although direct evidence is limited, the degradation mechanism of HMGR is highly conserved, suggesting that GGPP similarly promotes HMGR degradation primarily through post-translational regulation. As one of the final products of sterol biosynthesis, stigmasterol can inhibit HMGR activity, thereby regulating sterol homeostasis within plants (Russell and Davidson 1982). Although the precise molecular mechanism remains incompletely elucidated, research suggests stigmasterol may indirectly reduce HMGR activity by influencing the conformation of HMGR or acting synergistically with hormones such as ABA (Russell and Davidson 1982). In the *Zmcyp710a8* mutant, which is deficient in stigmasterol, the application of exogenous stigmasterol induces the mRNA expression of both *ZmHMGR* and *ZmSMT2* (Aboobucker et al. 2021). This seemingly polarized effect of stigmasterol may result from its differential influence on HMGR activity at varying concentrations, as well as its involvement in both transcriptional and post-transcriptional level of HMGR. Additionally, the responsiveness of HMGR to stigmasterol may exhibit variability across different plant species. In conclusion, the intricate regulation of HMGR by sterol intermediates and end products is essential for maintaining sterol homeostasis in plants.

### SQS

SQS serves a critical role as a branch point enzyme in plant metabolism, directing assimilated carbon flux towards the biosynthesis of triterpenes and sterols (Aminfar and Tohidfar 2018). This enzyme catalyzes a two-step reaction, initiating with the condensation of two FPP (C15) molecules into presqualene diphosphate (PSPP), followed by the reduction of PSPP to squalene, a process that utilizes NADPH as a cofactor (Nakashima et al*.* 1995, Pandit et al. 2000). The number of *SQS* members varies significantly across species, with a single gene present in yeast and humans (Robinson et al. 1993), while plants may contain one to three copies of *SQS* genes (Busquets et al. 2008). SQS is classified under the type I family, which is distinguished by the mechanism of carbocation formation via phosphate group dissociation (Harwood Jr et al. 1997). It shares mechanistic features with other class I isoprenoid biosynthetic enzymes, such as FPPS, which synthesizes the SQS substrate, 2-*trans*,6-*trans*-farnesyl diphosphate (Tsujimoto 1916). The presence of DDXXD aspartate-rich motifs is a commonality among these enzymes, facilitating substrate binding and subsequent phosphate group release in the presence of Mg^2+^ ions (Biller et al. 1996). Structurally, SQS resembles other isoprenoid biosynthetic enzymes, sharing an α-helical core that forms an inner channel and includes the highly conserved aspartate-rich region, which is of particular interest in the context of squalene synthase inhibitor studies. The enzyme's architecture is characterized by a C-terminal hydrophobic transmembrane domain that anchors SQS to the ER membrane, and a substantial catalytic N-terminal domain that extends into the cytosol (Stamellos et al. 1993).

Recently, researchers have made significant advancements in understanding *SQS* genes across various species, including *Atractylodes lancea* (Wu et al. 2022), *Camellia vietnamensis* (Dai et al. 2022), *Dioscorea nipponica* (Feng et al. 2023), and *Chrysanthemum morifolium* (Firsov et al. 2024). As an important enzyme in phytosterol biosynthesis, *SQS* is regulated by the *cis*-elements that sense stress and plant hormones. MeJA has the potential to function as an elicitor for the expression of the *SQS* gene in various plant species, including *Atractylodes lancea* (Wu et al. 2022), *Taraxacum antungense* (Liu et al. 2022; Liu et al. 2023b), and *Siraitia grosvenorii* (Mu et al. 2025). In *T. antungense*, MeJA can activate the bHLH transcription factor TaMYC2, which then increases the expression of the *TaSQS* gene by 3-5 fold (Liu et al. 2023b). Furthermore, a newly identified transcription factor, EsbZIP, related to the *SQS* gene has emerged (Li et al. 2024a). Plants synthesize various sterols, triterpenes, and secondary metabolites. The evolution of multiple *SQE* genes helps regulate distinct metabolic pathways in different tissues, developmental stages, and in response to environmental and hormonal stimuli.

### SQE

SQE catalyzes the oxidation of squalene to 2,3-oxidosqualene, a reaction that represents the first oxidation step in triterpene biosynthesis and involves the flavoprotein NADPH-cytochrome P450 reductase (CPR) (Lin et al*.* 2017). The regulable nature of this enzymatic step highlights its essential role in controlling sterol and triterpenoid synthesis. *SQE* is typically represented by a single gene in yeast and humans. Contrastingly, multiple paralogs of *SQE* can be found in some plant species (Rasbery et al. 2007; Han et al. 2010). These various isoforms likely serve distinct functions; for instance, in *A. thaliana*, only 3 out of 6 *SQE* isoforms can complement the yeast *erg1* mutant, restoring its wild-type activity (Rasbery et al. 2007). Also, among the 16 *BnSQEs* analyzed, only *BnaA01.SQE3*, *BnaC01.SQE3*, and *BnaA09.SQE3* exhibited upregulation following treatment with MeJA (Wang et al. 2024b).

SQE is localized to the ER in plants, tethered by transmembrane domains whose number may differ even within a single species (He et al. 2008; Han et al. 2010; Zhu et al. 2024). Moreover, the turnover and homeostasis of *AtSQE1* are mediated by non-targeted N-terminal acetylation via the AtDOA10s-like E3 ligase in the process of ERAD (Etherington et al. 2023). Research focusing on the *Celastrus orbiculatus SQE* gene (*CbSQE*) has demonstrated that its overexpression leads to the coordinated upregulation of genes involved in sterol and triterpene biosynthesis, both upstream (*SQS*) and downstream (*CYP51* and *OSCs*) of *SQE*. This indicates a regulatory role for *CbSQE* that extends beyond its immediate catalytic function, potentially influencing a network of biosynthetic pathways central to sterol and triterpenoid production (Kaushal et al. 2023). Furthermore, there is a newly identified TF named SgTCP24 in *Siraitia grosvenorii*, which could directly bind and activate *SgSQE* (Mu et al. 2025). These insights into the *SQE* gene and its encoded enzyme underscore its strategic influence on crucial metabolic pathways in plants, with significant implications for producing commercially and medically valuable phytochemicals.

### OSC

Triterpene structures in plants are formed through the cyclization of 2,3-oxidosqualene by enzymes belonging to the oxidosqualene cyclase (*OSC*) family. The various cyclization pathways and subsequent modifications result in the unique and diverse triterpene structures observed across the plant kingdom. To date, a total of 152 *OSCs* from different plant species have been identified, enabling the formation of 100 distinct triterpene scaffolds (Wang et al. 2022; Hakim et al. 2025). Among the most well-characterized OSCs are β-amyrin synthase (BAS), cycloartenol synthase (CAS), and lupeol synthase (LAS), which together account for the majority of known triterpenoid scaffolds. In hexaploid wheat (*Triticum aestivum*), members of the *OSC* gene family exhibit differentiated expression patterns across different tissues and developmental stages, demonstrating homeologous expression bias, where certain homologous genes show higher expression in specific tissues or developmental stages (Guo et al. 2022). For instance, Ta5A004900 and Ta5D011800 are specifically highly expressed in leaf sheaths and flag leaf sheaths, indicating that homologous genes from the A and D subgenomes dominate functional expression in these tissues (Guo et al. 2022). Homologous expression bias may be jointly regulated by multiple mechanisms, including differences in promoter *cis*-elements, epigenetic modifications, and transcription factor binding specificity (Guo et al. 2022). This expression bias is closely associated with subfunctionalisation, aiding polyploid plants in flexibly regulating triterpenoid metabolism under diverse environmental and physiological demands. In other plants, such as *Artemisia annua* (Guo et al. 2025) and *Astragalus membranaceus* (Chen et al. 2023), the *OSC* gene also exhibits tissue-specificity and stress-induced expression differentiation. However, direct reports on homologous expression bias are currently concentrated primarily in polyploid crops.

Plant *OSC*s are crucial for the biosynthesis of triterpenes and sterols, with their expression and activity undergoing intricate regulation at multiple levels: transcriptional, epigenetic, post-translational, and metabolic. At the transcriptional level, the promoter regions of *OSC*s are abundant in *cis*-elements that respond to light, hormones, and stress, exhibiting distinct distributions of these regulatory elements among different *OSC* subfamilies (Guo et al. 2025). A variety of transcription factors, such as MYB, NAC, WRKY, and ERF, can regulate *OSC* expression both positively and negatively (Cui et al. 2025). For instance, EsMYB4 and EsERF66 positively influence EsbAS1 while negatively regulating EsCAS1, whereas EsNAC047, EsNAC098, and EsWRKY40 demonstrate opposing regulatory effects. On the post-transcriptional level, the methylation status of promoters plays a critical role in influencing *OSC* expression activity (Cui et al. 2025). At the post-translational level, certain *OSC*s possess specialized domains, such as the F-box, indicating potential regulation of protein degradation through mechanisms like ubiquitination (Guo et al. 2025). Additionally, plant hormones like JA and ABA, along with UV-B radiation and pathogen-induced stress, significantly boost *OSC* expression, thereby modulating triterpenoid synthesis to strengthen resistance (Dhar et al. 2014; Jin et al. 2017; Guo et al. 2025). Overall, *OSC*s are subject to a sophisticated network of regulation at multiple levels, encompassing transcriptional, epigenetic, protein modification, plant hormones and stress signaling pathways. This multifaceted regulation is essential for maintaining a dynamic equilibrium and ensuring environmental adaptability in triterpenoid synthesis.

### SMT

Phytosterols exhibit a defining structural feature: a 24-alkyl group on the sterol side chain (Bouvier‐Navé et al. 1997). This group is synthesized by an enzyme known as *SMT*. *SMT*s are categorized into two distinct types based on their catalytic functions. The first, *SMT1*, introduces a methyl group at the C24 position via a series of reactions. The second, *SMT2*, adds an ethyl group to the C24 side chain through a similar sequential process. Specifically, *SMT2* is responsible for catalyzing the transformation of 24-ethylidene lophenol into 24-methylene lophenol in plant species. On the other hand, *SMT1* is known to encode cycloartenol-C24-methyltransferase (Bouvier‐Navé et al. 1998). Functional analyses of *SMT*s have been conducted in model organisms such as *Nicotiana tabacum* and *Arabidopsis thaliana* (Schaeffer et al. 2000). Recent advancements have included the isolation and characterization of *TwSMT1* cDNA from *Tripterygium wilfordii*, confirming the methyltransferase activity of SMT1 (Guan et al. 2017).

The expression of *SMTs* was affected by plant organs, growth status, and abiotic stress. At the vegetative stage, *SMTs* are primarily involved in cell elongation and expansion. In soybeans, the expression of *SMT1* was higher in younger leaves, roots, and stems than in mature ones. It decreased sharply after the formation of young pods and immature seeds (Shi et al. 1996). The younger stage of Arabidopsis rosette (Diener et al. 2000), *W. somnifera* root, leaves, and stem (Pal et al. 2019), and *Paris polyphylla* root (Guan et al. 2018) all exhibited higher *SMT1* expression. Tissue expression pattern analysis in *T. wilfordii* revealed significantly higher expression of *TwSMT1* in the phellem layer compared to the leaf, stem, xylem, and phloem organs (Guan et al. 2017). In addition to the growth stage and expression site, abiotic stresses are also important factors affecting the expression of *SMTs*. The sequencing of the *TaSMT1* (*Triticum aestivum*) promoter region identifies that *cis*-elements are involved in low-temperature response. These *cis*-elements, in conjunction with other factors, determine the differential effects of stress on the expression of homoeologous *TaSMT1* genes. For instance, *TaSMT1*−5A is expressed constitutively in both roots and leaves, while the *TaSMT1*−4D gene responds highly to cold stress (Renkova et al. 2019).

### SMO

Three separate enzymatic reactions are involved in each C-4 demethylation step. A *SMO*, a 3-hydroxysteroid-dehydrogenase/C4-decarboxylase (*3βHSD/D*) (Rondet et al. 1999), and a 3-ketosteroid reductase (*SR*) are involved in this process (Pascal et al. 1994). Two different sterol methyl oxidases, *SMO1* and *SMO2*, have evolved in *Arabidopsis,* and they function in a non-consecutive manner with three and two isoforms, respectively (Darnet and Rahier 2004). On the other hand, only one *SMO* enzyme has been identified in both fungi and mammals, which is involved in the C4-demethylation enzyme complex (Darnet et al. 2021). With the help of other enzymes, SMO1 and SMO2 in plants remove the methyl group at the C4 position by using 24-Methylenemethylene-cycloartanol (Bouvier‐Navé et al. 1997) and 24-Ethylideneethylidene-lophenol (Darnet and Rahier 2004) as substrates, respectively. As an indispensable enzyme in the phytosterol biosynthetic pathway, SMO1-1 has been proven to interact with acyl-coenzyme A-binding proteins (ACBP), which are involved in fatty acid and sterol metabolism. Upon overexpression or mutation of *acyl-CoA-binding protein 1 (ACBP1)* and/or *SMO1-1*, there are quantitative and compositional changes in fatty acids and sterols (Lung et al. 2017, 2018). Research by Lung et al. (2017) shows that Arabidopsis *ACBP1* regulates sterol synthesis by interacting with *SMO1*-*1*. Unlike *SMO1-1*, silencing *SMO1-2* in *acbp1* mutants impairs seed development and gamete transport, as well as pollen function (Lung et al. 2018).

As crucial enzymes in phytosterol biosynthesis, SMOs could affect diverse kinds of plant hormones to manipulate the embryo development and stress tolerance of plants. Three Arabidopsis *SMO1* members have been identified (Song et al. 2019). Single *smo1* mutants or *smo1-1 smo1-3* double mutants did not affect phenotype. However, it was discovered that *smo1-1 smo1-2* double mutants were embryo-lethal due to abnormal expression of auxin biosynthesis and response reporters, and reduced cytokinin biosynthesis and response (Song et al. 2019). And the same situation could be found in *smo2-1 smo2-2* double mutant (Zhang et al. 2016), and these two genes both could regulate Arabidopsis embryogenesis and postembryonic development. *SMO* not only controls the balance of auxin and cytokinin, but it can also be regulated by plant hormones. *Artemisia annua* is a plant that can produce the anti-malarial drug artemisinin. According to the research of Alka Singh et al. (2015), *AaSMO1* expression was significantly induced by osmotic and dehydration stress, and its promoter contained an ABA responsive element.

## Biotechnology to increase phytosterols in plants

**Fig. 2:**
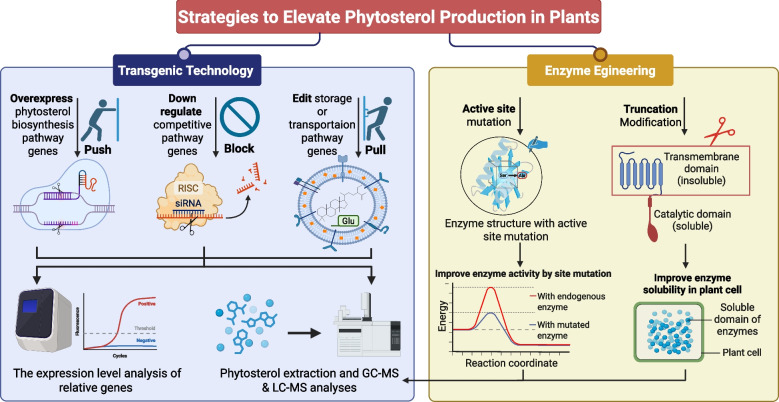
Strategies to elevate phytosterol production in the plant system. The schematic summarizes two complementary approaches. Left panel (Transgenic technology) uses a “push–block–pull” framework: push by overexpressing key genes in the phytosterol biosynthesis pathway; block by downregulating competing pathways; and pull by editing storage and transport genes. Right panel (Enzyme engineering) shows two routes: active‑site mutation to increase catalytic efficiency and lower the reaction’s activation energy (illustrated by a reduced barrier on a reaction‑coordinate curve), and truncation to remove insoluble transmembrane domains so that the soluble catalytic domain functions more effectively in plant cells. Together, these interventions increase metabolic flux and enhance phytosterol yield. Created in https://BioRender.com

The mainstream techniques for enhancing the biosynthesis of secondary metabolites, such as phytosterols, are gene editing and metabolic engineering (Fig. 2). By overexpressing or knocking out key enzyme genes, the flux of biosynthetic pathways can be amplified, thereby increasing sterol accumulation. CRISPR/Cas technology stands as one of the most revolutionary gene editing tools currently available, enabling highly efficient and specific editing at designated genomic sites, including gene knockout, knock-in, and base substitution. It can edit not only single genes but also enable simultaneous multi-gene editing, making it suitable for complex trait improvement and metabolic pathway reconstruction. Enzyme evolution, meanwhile, improves post-transcriptional enzyme activity by modifying the active site. However, while targeted modification of plant metabolic pathways can significantly increase yields, off-target effects, gene transformation efficiency, and complex regulatory networks remain challenges to be addressed.

### Transgenic technology

The enhancement of plant nutrient biosynthesis usually involves metabolic engineering techniques known as "push" (which increases the flow of biosynthesis), "block" (which suppresses competing pathways), and "pull" (which encourages the storage and transport of metabolites) (Perez‐Colao et al. 2025). Plant biotechnology can be used to produce high levels of phytosterols by employing these strategies (Fig. 2). The primary tactic is often to overexpress genes encoding the rate-limiting enzymes. Different enzymes in the biosynthetic pathway have been targeted to increase phytosterol levels: HMGS, HMGR, FPPS, SQS, SQE, OSC, SMT, and SMO (Table 1). Alternatively, the overexpression of specific transportation- or storage-related genes can also be used to stimulate the accumulation of phytosterols. In *Arabidopsis thaliana*, the overexpression of *HMGR1* leads to significant increases in sterol levels, concomitant with the accumulation of specific intermediates within the phytosterol biosynthetic pathway (Lange et al. 2020). This phenomenon is explicable, as HMGR functions at the initial stages of this biosynthetic pathway. The correlation between elevated expression levels of *HMGR* and increased phytosterol concentrations has been substantiated in various species, including *Hevea brasiliensis* (rubber tree) (Harker et al. 2003), *Glycine max* (Wang et al. 2023a), *Lavandula latifolia* (lavender) (Muñoz‐Bertomeu et al. 2007), and *Digitalis minor* (Sales et al. 2007). Notably, individual mutations in the *HMGR* gene may not yield significant increases in phytosterol levels due to the stringent regulatory mechanisms governing phytosterol biosynthesis (Table 1). For instance, the overexpression of *HMGR* in *Thalassiosira pseudonana* does not correlate with enhanced phytosterol accumulation (Jaramillo-Madrid et al. 2020). Therefore, it is proposed that co-expression of *HMGR* alongside a downstream enzyme involved in phytosterol synthesis could strategically enhance metabolic flux toward phytosterol production. The co-overexpression of *PnHMGR* and *PnSS* in *Panax notoginseng* significantly alters the biosynthesis of triterpene saponins and phytosterols, resulting in approximately 1.8 times and 2.0 times higher levels of β-sitosterol and stigmasterol, respectively, compared to wild-type cells (Deng et al. 2017). In *Paris polyphylla*, the co-expression of the *PpHMGR* and *PpOSC1* genes has facilitated the attainment of elevated cycloartenol levels, reaching 28.79 mg/g dry weight (Yin et al. 2023). Furthermore, *HMGR* can be co-expressed with various downstream biosynthetic enzymes that target specific phytosterols. Notably, the co-overexpression of genes encoding enzymes such as *SMO1* or *SMT1* with *HMGR1* can alleviate metabolic flux constraints, thereby promoting the enhanced production of sterol end products (Lange et al. 2020). In rice, the co-expression of *tHMGR* and other MVA pathway genes *Brassica juncea HMGS* (*BjHMGS*)*, Catharanthus roseus* mevalonate kinase (*CrMK*)*, C. roseus* 5-phosphomevalonate decarboxylase (*CrPMK*)*, C. roseus* mevalonate decarboxylase (*CrMVD*) along with the transcription factor WRINKLED 1, led to a fivefold upregulation of early genes in the MVA pathway (*OsHMGS* and *OsHMGR*). Meanwhile, initiation and termination genes of the MEP pathway 1-deoxy-D-xylulose 5-phosphate reductoisomerase (*OsDXR*), 2-*C*-methyl-D-erythritol 4-phosphate cytidylyltransferase (*OsMDS*), 4-hydroxy-3-methylbut-2-enyl diphosphate synthase (*OsHDS*), 4-hydroxy-3-methylbut-2-enyl diphosphate reductase 1 (*OsHDR1*), IPP isomerase 1 (*OsIPPI1*) were downregulated over twofold. As a result, campesterol levels increased by 1.41 to 2.24-fold in 25% of transgenic lines compared with wild tpye, stigmasterol levels rose by 1.53 to 3.56-fold in 50% of lines, β-sitosterol levels climbed by 1.32 to 2.92-fold in 58% of lines, and cycloartenol levels surged by 1.91 to 7.20-fold in 58% of lines (Pérez et al. 2022).

**Table 1:** Metabolic engineering of phytosterols in plants (2019–2025)

Host Species	Tissue	Genes	Gene Edit Type	Other Affected Genes	Result				References
					Stigmasterol	β-sitosterol	Campesterol	Total sterol	
*A. thaliana*	Leaf	*GmHMGR4*	Overexpression	*	↑	↑	↑	*	Wang et al. 2023a
*A. thaliana*	Leaf	*GmHMGR6*	Overexpression	*	↑	↑	↑	*	Wang et al. 2023a
*P. polyphylla*	Leaf	*PpHMGR, PpOSC1*	Overexpression	*	*	*	*	↑	Yin et al. 2023
*O. sativa*	Endosperm	*tHMGR* *BjHMGS* *CrMK* *CrPMK* *CrMVD* *WRINKLED1*	Overexpression	*OsHMGS* ↑ *OsHMGR* ↑	↑	↑	↑	↑	Pérez et al. 2022
				*OsDXR* ↑ *OsMDS* ↑ *OsHDS* ↓ *OsHDR1* ↓ *OsIPPI1* ↓					
*A. thaliana*	Seed	*TgSQS*	Overexpression	*	–	↑	–	↑	Zhang et al. 2023
*N. benthamiana*	Leaf	*NbSQS*	Overexpression	*	↑	–	–	*	Fu et al. 2023
*N. tabacum*	Leave	*CbSQE*	Overexpression	*CbSQS* ↑ *CbCYP51* ↑ *CbOSCs* ↑	↑	*	*	*	Kaushal et al. 2023
*P. ginseng*	Root	*PgSQE1*	Overexpression	*PgDDS* ↑ *PgCAS* ↑	↑	↑	↑	↑	Han et al. 2020
*N. tabacum*	Root & Shoot	*GgSQE1*	Overexpression	*	↑	↑	↑	↑	Manzoor et al. 2021
*N. tabacum*	Leaf	*TcOSC1*	Overexpression	*	↓	↓	↓	*	Han et al. 2022)
*T. coreanum*	Leaf	*TcOSC1*	Overexpression	*	↓	↓	↓	*	Han et al. 2022
*N. benthamiana*	Leaf	*PpOSC1*	Overexpression	*	*	*	*	↑	Hua et al. 2022
*W. somnifera*	Leaf	*WsSMT1*	Downregulation	*	↓	↓	↓	*	Pal et al. 2019
*N. tabacum*	Leaf	*WsSMT1*	Overexpression	*	↑	↑	↑	*	Pal et al. 2019
*G. hirsutum*	Fiber	*GhSMT2-1*	Overexpression	*	*	↑	↓	*	Niu et al. 2019
*G. hirsutum*	Fiber	*GhSMO2-2*	Overexpression	*	↑	↑	↑	↑	Liu et al. 2023a
*D. lanata*	Shoot	*GlSMO1*	Overexpression	*	*	*	*	↑	Raghavan et al. 2023,
*O. sativa*	Seed	*OsSMO1*	Knockout	*	↓	↓	↓	↓	Lu et al. 2025a
*A. thaliana*	Seed	*OsSCYL2*	Overexpression	*	↑	↑	↑	*	Xu et al. 2024
*P. trifoliata*	Shoot	*PtrERF110*	Overexpression	*PtrUGT80B1* ↑	*	*	*	↑	Khan et al. 2024

This table summarizes sterol composition shifts induced by genetic manipulation of phytosterol-related genes across different host species and tissues. ↑: increase; ↓: decrease; –: no change; *: data unavailable or not directly comparable. *A. thaliana*: *Arabidopsis thaliana*; CAS: cycloartenol synthase; CYP51: sterol 14α-demethylase; *D. lanata*: *Digitalis lanata*; DDS: dammarenediol-II synthase; DXR: 1-deoxy-D-xylulose 5-phosphate reductoisomerase; ERF110: ethylene-responsive transcription factor 110; *G. hirsutum*: *Gossypium hirsutum*; HDR: 1-hydroxy-2-methyl-2-butenyl 4-diphosphate reductase; HDS: 1-hydroxy-2-methyl-2-butenyl 4-diphosphate synthase; HMGR: 3-hydroxy-3-methylglutaryl-coenzyme A reductase; HMGS: 3-hydroxy-3-methylglutaryl-coenzyme A synthase; IPPI: isopentenyl diphosphate isomerase; MDS: 2-*C*-methyl-D-erythritol 2,4-cyclodiphosphate synthase; *N. benthamiana*: *Nicotiana benthamiana*; *N. tabacum*: *Nicotiana tabacum*; *O. sativa*: *Oryza sativa*; OSC: oxidosqualene cyclase; *P. ginseng*: *Panax ginseng*; *P. polyphylla*: *Paris polyphylla*; *P. trifoliata*: *Poncirus trifoliata*; *SCYL2*: SCY1-like protein kinase 2; SMO: sterol C-4 methyl oxidase; SMT: sterol C-24 methyltransferase; SQE: squalene epoxidase; SQS: squalene synthase; *T. coreanum*: *Taraxacum coreanum*; UGT80B1: UDP-glucose:sterol glucosyltransferase 80B1; *W. somnifera*: *Withania somnifera*

While these findings highlight the potential of HMGR overexpression and co-expression strategies for enhancing phytosterol production, critical gaps remain in translating lab-scale successes to practical applications. The long-term effects of metabolic engineering on plant growth, stress tolerance, and overall fitness are rarely evaluated, which is essential for sustainable agricultural deployment.

It has been defined above that overexpression of *SQS* has been shown to elevate the biosynthesis of various phytosterols (Table 1). For instance, stable overexpression of *SQS* in *Nicotiana benthamiana* leaves not only compromised resistance to *Phytophthora infestans* but also resulted in a 160% increase in stigmasterol accumulation compared to the control, while sitosterol and campesterol levels remained unaffected (Fu et al. 2023). In the study by Zhang et al., *Arabidopsis thaliana* was engineered to express the *SQS* gene from *Torreya grandis* under the control of a strong CaMV35S promoter. The transgenic lines exhibited a 15% increase in β-sitosterol content in mature seeds relative to the wild type, whereas campesterol levels did not significantly change (Zhang et al. 2023). Additionally, the overexpression of the *SQS* gene from *Torreya grandis* (*TgSQS*) in Arabidopsis led to increased levels of β-sitosterol and squalene (Zhang et al. 2023) highlighting the gene's influence on phytosterol biosynthesis. Although there is some progress that has been made by manipulating the *SQS* gene, *SQS* is located relatively upstream of sterol biosynthesis. Other strategies, such as co-expression with downstream biosynthesis genes, could be considered.

SQE is another critical rate-limiting enzyme in phytosterol biosynthesis (Table 1). It was demonstrated that the overexpression of *CbSQE* in transgenic plants led to elevated expression levels of genes both upstream and downstream in the biosynthetic pathway, namely *CAS*, *βAS*, and cytochrome P450 monooxygenase 51 (*CYP51*), suggesting that SQE exerts a regulatory influence over phytosterol biosynthesis (Manzoor et al. 2021). Transgenic *Panax ginseng* roots overexpression of *PgSQE1* significantly upregulated the expression of key biosynthetic enzymes, dammarenediol-II synthase (PgDDS) and cycloartenol synthase (PgCAS), thereby boosting ginsenoside and phytosterol production (Han et al. 2020). Liquid chromatography-ion trap-time of flight mass spectrometry (LC-IT-TOF–MS) analysis revealed that total ginsenoside content in the adventitious roots of *PgSQE1*-overexpressing transgenic lines was at least 50% higher than in the wild type. Similarly, gas chromatography-mass spectrometry (GC–MS) analysis indicated a significant increase in sterol content in these transgenic plants compared to the wild type. Constitutive expression of *GgSQE1* from *Glycyrrhiza glabra* in tobacco also modulated phytosterol content (Manzoor et al. 2021). Extensive research on the *Celastrus orbiculatus SQE* gene (*CbSQE*) has revealed that its overexpression elicits a harmonious upregulation of genes pivotal for sterol and triterpene biosynthesis, extending both upstream to *SQS* and downstream to *CYP51* and *OSCs* in the biosynthetic cascade (Kaushal et al. 2023). While these studies underscore the pivotal role of SQE in coordinating phytosterol biosynthesis through trans-regulatory effects on upstream and downstream genes, critical gaps remain in translating these findings to practical applications. The molecular mechanisms by which SQE overexpression orchestrates the coordinated upregulation of biosynthetic genes are poorly characterized, whether through direct protein–protein interactions or indirect signaling cascades remain unclear.

*OSCs* are also regarded as a strategic target to enhance phytosterol biosynthesis (Table 1). However, overexpression of *OSCs* does not always yield the desired outcomes. *TcOSC1* from *Taraxacum coreanum* functions as a versatile oxidosqualene cyclase, facilitating the synthesis of various triterpenes. Despite the production of multiple triterpenes in *TcOSC1*-transgenic tobacco, a reduction in phytosterol content (β-sitosterol, campesterol, and stigmasterol) was observed. *TcOSC1* competes vigorously for 2,3-oxysqualene in synthesizing multiple triterpenes (Han et al. 2022). This competition restricts the flow toward sterol synthesis via the cycloartenol synthase pathway, ultimately leading to a decrease in total sterol production. This underscores the necessity of careful OSC selection for overexpression to avoid unintended shifts in metabolite profiles. Following the infiltration of *A. tumefaciens* transformed with the *PpOSC1* gene into *N. benthamiana*, cycloartenol levels in the leaves demonstrated an increase of 3.12-fold. This enhancement in cycloartenol may subsequently contribute to an elevated phytosterol content (Hua et al. 2022). At the fork junction of phytosterol biosynthesis pathway, OSCs act as train switches that determine the direction of carbon flux. OSC isoforms selection and modulation of their expression levels represent key metabolic engineering strategies to enhance phytosterol accumulation in plants.

Plants have a distinct structural characteristic in their sterols, which includes a 24-alkyl group present in the sterol side chain (Bouvier‐Navé et al. 1997). This group is synthesized by an enzyme known as *SMT*. In cotton, the overexpression of *GhSMT2-1* led to an increase in sitosterol content, while simultaneously reducing the campesterol/brassicasterol ratio and lowering campesterol levels. Transgenic cotton fiber cells exhibited shorter and thicker fibers, suggesting that the altered phytosterol composition affected cell growth and wall deposition (Niu et al. 2019). In *Withania somnifera*, RNAi-mediated suppression of *SMT1* resulted in decreased levels of campesterol, sitosterol, and stigmasterol, accompanied by an increase in cholesterol content (Pal et al. 2019). By employing gene editing techniques on *SMT* genes, it is possible to significantly modify the content and composition of phytosterols in various plants (Table 1). These modifications not only affect plant growth and development but can also be leveraged to improve crop quality and enhance stress tolerance.

With the widespread application of gene-editing technologies such as CRISPR/Cas9, scientists have begun attempting to regulate phytosterol content within plants by editing *SMO* and its homologues (Table 1). *AaSMO1* converts 4,4-dimethylsterol into 4α-methylsterols in *Artemisia annua*. Overexpressing *AaSMO1* boosts both substrate and product levels, along with an increase in sitosterol and stigmasterol in transgenic lines (Singh et al. 2015). Currently, no direct interaction between SMO and HMGR has been confirmed; this phenomenon is likely due to feedback from downstream phytosterol intermediates that enhance HMGR activity to maintain carbon flow in the sterol pathway. In *Digitalis lanata*, *SMO1* gene expression is positively correlated with phytosterol synthesis, and exogenous induction of *SMO1* expression enhances phytosterol levels (Raghavan et al. 2023). *GhBES* is a transcription factor in the BR signaling pathway in upland cotton (*Gossypium hirsutum* L.). It can regulate the transcription of *GhSMO2* by directly binding to its promoter. Furthermore, the increased expression of *GhSMO2-2* notably enhanced the elongation of fiber cells and the production of phytosterols in cotton, as well as raised the levels of certain sphingolipid species that are crucial for fiber elongation (Liu et al. 2023a). Downregulation of *SMO* genes could also affect phytosterol and its intermediates levels. Lu et al. (2025a) harnessed CRISPR-Cas9 to meticulously target and silence the *OsSMOs* gene in rice. In comparison to the WT, the *ossmo2-1* strain demonstrated a significant reduction in the levels of campesterol, sitosterol, and stigmasterol. Interestingly, this mutant also showed a noteworthy increase in the precursor compound 24-ethylidenelophenol, while also exhibiting a decline in the downstream product Δ^7^-avenasterol (Lu et al. 2025a). These findings suggest that *OsSMO2-1* is a key player in the biosynthesis of sitosterol and stigmasterol, highlighting its importance in this metabolic pathway (Lu et al. 2025a).

Except for the key biosynthetic genes, the gene related to metabolite transportation and storage could also induce phytosterol production (Table 1). The *O. sativa SCY1-LIKE2* (*OsSCYL2*) gene, characterized by its *SCY1*-like properties, has been identified as an integral component of clathrin-coated vesicles, playing a pivotal role in the clathrin-mediated vesicular trafficking processes associated with the Golgi apparatus (Yao et al. 2022). Experimental observations have indicated that the overexpression of *SCYL2* in rice cultivars leads to an increased accumulation of phytosterols such as campesterol, stigmasterol, and β-sitosterol. Conversely, a marked reduction in these sterols was noted in the *scyl2* mutant (Xu et al. 2024). Sterol glycosyltransferase transfers glucose (UGT) from UDP-glucose to the C-3 hydroxyl group of sterols, producing steryl glucosides (SG) and acylated steryl glucosides (ASG). This process increases the ratio of free to conjugated sterols, essential for regulating membrane structure, development, and stress responses (He et al. 2022). The cold-induced transcription factor *PtrERF110* in *Poncirus trifoliata* directly activates genes such as *UGT80B1*, thereby increasing plant sterol content and enhancing cold tolerance (Khan et al. 2024). However, the specific sterol content across different tissues and plant varieties was not examined in this study. This empirical evidence underlines the potential of leveraging enhanced sterol transportation and storage as a strategic avenue for augmenting the metabolic flux within plant phytosterol biosynthetic pathways.

The scalability of these strategies to industrial production systems faces challenges such as cost-effective transformation methods and maintaining stable transgene expression. Future research should address these gaps by integrating multi-species comparative analyses, life-cycle assessment of engineered plants, and optimization of expression systems for large-scale production.

### Enzyme engineering

The biosynthesis of phytosterols involves a complex network of enzymes, and recent advances in enzyme engineering have significantly enhanced the production and manipulation of these critical enzymes, offering new avenues for optimizing sterol synthesis. A key strategy in enzyme engineering is the alteration of amino acids within the active site to modify enzyme substrate specificity or efficiency.

HMGS catalyzes the condensation of acetyl-CoA and acetoacetyl-CoA to produce HMG-CoA. Site-directed mutagenesis of the key motif in plant HMGS significantly boosts phytosterol content, demonstrating superior efficacy compared to the overexpression of wild-type HMGS. In *Brassica juncea*, the S359A mutant (Serine 359 → Alanine) of HMGS1 shows a remarkable tenfold increase in enzyme activity. When introduced into plants such as Arabidopsis (Wang et al. 2012), tobacco (Liao et al. 2014), and tomato (Liao et al. 2018), this mutant elevates total leaf phytosterol content by 20–30%, surpassing levels observed with wild-type wtOE-HMGS1. The S359A mutation modifies the enzyme's catalytic motif, bringing its scaffold closer to the catalytic loop and thereby accelerating reaction rates (Liao et al. 2018, 2021). This mutant not only enhances HMGS activity but also upregulates the expression of downstream sterol synthesis-related genes, including *HMGR*, *SQS*, and *SMT*, further facilitating phytosterol synthesis (Wang et al. 2012; Liao et al. 2018). While other variants, such as the H188N/S359A double mutant, also exhibit high enzyme activity and phytosterol-enhancing potential, the effect of the S359A mutation is particularly pronounced (Wang et al. 2012).

The process of mevalonate synthesis through HMG-CoA reductase is an indispensable step in the production of isoprenoids in plants. Site-directed mutagenesis, or the targeted modification of the plant HMGR motif, can significantly enhance phytosterol content, especially in seeds and leaves. Research has shown that the removal of the N-terminal membrane-binding domain of HMGR, resulting in a truncated version that retains only the catalytic domain, leads to a substantial increase in phytosterol levels in tobacco seeds and leaves. Specifically, seed phytosterol content increased by 3.2-fold, while the leaf content surged tenfold, accompanied by a notable accumulation of the intermediate cycloartenol (Harker et al. 2003). This approach continues to be widely utilized in heterologous expression across various plant species, including *N. benthamiana* (Romsuk et al. 2022) and *P. polyphylla* (Zhao et al. 2025). Additionally, optimizing subcellular localization (Zhao et al. 2025) and refining expression vectors, such as the Tsukuba system (Romsuk et al. 2022), can further enhance the efficiency of expression and yield of truncated HMGR. Another investigation targeted a key serine residue at the SnRK1 phosphorylation site of HMGR, effectively abolishing its phosphorylation regulation. The resulting mutant (AtHMG1m) exhibited a 2.44-fold increase in phytosterol content in tobacco seeds, markedly surpassing both the wild type and other controls (Hey et al. 2006). However, some mutants resulted in abnormalities in floral organ development or reduced seed set rates (Hey et al. 2006). Therefore, tissue-specific expression strategies are necessary for practical applications.

In recent years, there has been a growing focus on the active site optimization of phytosterol synthase genes, recognizing their critical role in determining terpenoid structures. Research on other genes for phytosterol biosynthesis has contributed to understanding enzyme active site dynamics. In Fritillaria, SQE enzyme evolution resulted in four mutations (C236R, M489L, G510A, and K517R) (Lu et al. 2020). Molecular docking analysis revealed that residue 236 is central within the binding pocket, while the other three residues are on the protein surface. Functional analysis demonstrated that the SQE (C236R) mutation significantly increased enzyme activity, as did the M489L and G510A mutations, which enhanced protein surface hydrophobicity (Lu et al. 2020). However, the evolution of SQE enzymes to enhance phytosterol production remains an uncharted territory.

OSCs have been the primary focus of active site research in phytosterol biosynthesis due to their significant contribution to triterpenoid motifs. Different OSCs determine their substrate specificity and product diversity through specific amino acid motifs. The underlying mechanisms manifest crucial roles in reaction initiation, substrate binding, product specificity, and carbocation stability. A structure–function relationship is evident in plant OSCs, with several conserved motifs identified, including the DCTAE sequence, which initiates the polycyclization sequence; the MXCXCR sequence, which affects product specificity; and the QXXXXXW patterns that help stabilize carbocation intermediates during cyclization (Chen et al. 2021). Chen et al. (2023) identified amino acid residues, VFM and VFN, as potential signature motifs for β-amyrin and cycloartenol synthases, respectively. And by substituting the relevant motifs in AmOSC2/3, TcOSC1, and TkOSC6 with VFN, the yield of cycloartenol increased by as much as 12.8-fold. Research on the role of individual amino acids has confirmed that the Tyr410 residue in both KcCAS and RsCAS enhances catalytic activity at this position (Basyuni et al. 2021). In AmOSC2, three residues, including Val727, Phe728, and Met729, are crucial for substrate stabilization, with Phe's cation-π interactions being particularly critical to the enzyme's catalytic function. For HcOSC6 in *Hemsleya chinensis*, three key amino acid residues, E246, M261, and D490, have been shown to stabilize intermediates and influence catalytic efficiency in cyclization reactions (Li et al. 2023). By analyzing the gene structure and conserved motifs of the *PaOSCs* (*Phellodendron amurense OSC*) gene family, Zhang et al. found that the insertion of long terminal repeat retrotransposons significantly contributes to the structural variation of *PaOSC* introns (Zhang et al. 2024). Diverse key residues in the catalytic center direct the substrate 2,3-oxidosqualene to prefold into different intermediate configurations. These intermediates can subsequently generate unique triterpenes through several stages of hydride and methyl group rearrangement, culminating in a final deprotonation reaction (Ma et al. 2024). This relationship not only underscores the evolutionary dynamics of the OSC family but also highlights the mechanisms driving genetic diversity and potential impacts on OCS function and adaptation in the organism. While most strategies aimed at enhancing phytosterol content typically focus on precursor synthases, such as HMGR and SQS, or on expression regulation, site-directed mutagenesis of motifs and protein engineering have surfaced as innovative approaches for increasing the concentration of specific products, including phytosterols.

## Heterologous production of phytosterols and their derivatives

**Fig. 3:**
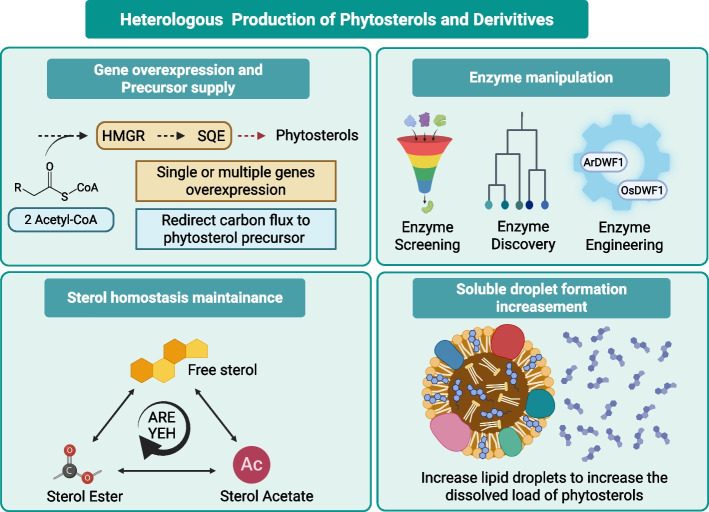
Heterologous production of phytosterols and derivatives. The diagram has four panels that outline an integrated design strategy for non‑native hosts. Top left (Gene overexpression and precursor supply): redirect carbon from acetyl‑CoA toward phytosterol precursors by overexpressing single or multiple pathway genes. Top right (Enzyme manipulation): combine enzyme screening, discovery, and protein engineering to identify high‑activity catalysts. Bottom left (Sterol homeostasis maintenance): balance pools of free sterol with sterol esters and sterol acetates. Bottom right (Soluble droplet formation increase): promote lipid‑droplet biogenesis to provide a soluble reservoir that sequesters and dissolves phytosterols, mitigating toxicity and increasing total product titers. ARE: Acyl-coenzyme A:sterol acyltransferase-related enzyme; ArDWF1: *Arabidopsis thaliana* DWARF1; HMGR: Hydroxymethylglutaryl-Coenzyme A Reductase; OsDWF1: Oryza sativa DWARF1; SQE: Squalene Epoxidase; YEH: Yeast Ester Hydrolase. Created in https://BioRender.com

Genetic engineering of microbial systems is a powerful tool for producing valuable specialized metabolites (Fig. 3). In yeast, for instance, manipulating key genes to redirect metabolic flux has successfully accelerated the synthesis of plant-derived phytosterols, highlighting the transformative potential of this field (Borodina and Nielsen 2014; Xu and Li 2020; Volk et al. 2022). While the native biosynthetic pathways of major phytosterols are well-elucidated in plants, metabolic engineering of microbial chassis can significantly enhance their yields far beyond what is achievable in their native hosts (Gu et al. 2021). The core principle of this approach, particularly in yeast, involves re-routing the endogenous ergosterol pathway toward the synthesis of target sterols. This typically requires a multi-pronged strategy encompassing precursor supply enhancement, pathway redirection, and optimization of cellular homeostasis (Meadows et al. 2016; Zhou et al. 2019; Liu et al. 2025; Qu et al. 2025).

### Enhancing precursor supply and co-expressing pathway modules

A primary strategy for boosting phytosterol production is to increase the availability of universal precursors. This often involves overexpressing key genes in upstream pathways, such as HMGR and SQE (Jaramillo-Madrid et al. 2020). More advanced approaches involve system-level rewiring of central carbon metabolism to maximize the flux towards the primary building block, acetyl-CoA, squalene and following phytosterols (Table 2) (Lin et al. 2023b). This was a critical step in a recent study that achieved record-breaking campesterol production (Liu et al. 2025), a success that powerfully demonstrates that a robust precursor supply is the foundation for high titers (Table 2).

**Table 2:** Summary of engineering strategies and production metrics for heterologous phytosterols

Strategic category	Key chassis organism(s)	Focus of strategy	Achievement/Production level	References
Precursor supply and module co-expression	*Saccharomyces cerevisiae;* *Escherichia coli*	Boosting raw materials (acetyl-CoA, squalene)	Building a "foundation" for high titers; achieving record campesterol flux	Jaramillo-Madrid et al. 2020; Lin et al. 2023b), Liu et al. 2025; Song et al. 2020; Takemura et al. 2017
Overcoming enzymatic bottlenecks	*Nicotiana benthamiana*	Transient library synthesis for standards	Customized analytical standard mixtures	Lu et al. 2025a
	*Saccharomyces cerevisiae*	Screening superior homologs (*DHCR7*)	Diagnosing and clearing "metabolic traffic jams"	Qian et al. 2020
	*Saccharomyces cerevisiae*	Protein engineering (*DWF1*)	Bypassing inefficient steps (*DWF1*)	Takemura et al. 2017; Jiang et al. 2023
Managing sterol homeostasis	*Saccharomyces cerevisiae*	Balancing storage vs. toxicity (push-and-pull system)	2.76 g/L (24-epi-ergosterol) 5.88 g/L (campesterol)	Jiang et al. 2023 Liu et al. 2025
Systems-level engineering	*Saccharomyces cerevisiae*	Integrating lipid droplet regulators with optimized promoters and fermentation conditions	1.44 g/L (campesterol in 5 L bioreactor)	Qin et al. 2024
Advanced applications	*Yarrowia lipolytica*	Creating specific "functional blends" (e.g., for bees)	Functional success (measured by bee brood health, not just titer)	Moore et al. 2025

This table shows the hierarchical metabolic engineering strategies employed to optimize the biosynthesis of phytosterols in various microbial and plant chassis. The strategic categories represent a systematic approach to pathway optimization. First, “Precursor supply and module co-expression” establishes the foundational carbon flux toward universal precursors such as squalene and acetyl-CoA. Second, “Overcoming enzymatic bottlenecks” details the resolution of pathway inefficiencies through the development of bespoke analytical standards in transient hosts and the identification of superior enzyme homologs. Third, “Managing sterol homeostasis” highlights the transition to high-titer production (up to 5.88 g/L) by implementing "push-and-pull" dynamics to mitigate cellular toxicity and balance sterol storage. Fourth, “Systems-level engineering” integrates these genetic interventions with bioreactor optimization and lipid droplet regulation to achieve industrial-scale yields. Finally, “Advanced applications” illustrates the shift from single-molecule titers to the rational design of functional multi-component sterol blends for ecological engineering

Beyond single-gene overexpression, co-expression of multiple enzymes is crucial. This can range from assembling cassettes for cycloartenol production in *Escherichia coli* (Takemura et al. 2017) to ensuring the presence of necessary redox partners for enzymes like SQE (Song et al. 2020), which is vital for reconstituting functional biosynthetic pathways of certain compounds, including phytosterols in heterologous hosts.

### Overcoming enzymatic bottlenecks and pathway inefficiencies

A key challenge in heterologous systems is the emergence of metabolic bottlenecks, leading to the accumulation of undesired or dead-end intermediates. Diagnosing the precise location of these bottlenecks is often hindered by a critical analytical gap: the scarcity of commercially available standards for many phytosterol pathway intermediates.

To address this analytical gap, Lu et al. (2025a) developed an innovative strategy. They employed *Nicotiana benthamiana* as a transient expression host for the in vivo synthesis of a library of phytosterol intermediates. These compounds were subsequently characterized by GC–MS and pooled to create a "customized standard mixture" (Lu et al. 2025a). The diagnostic utility of this bespoke tool was then validated through the analysis of rice mutants (*ossmo2-1*), revealing a significant accumulation of 24-ethylidenelophenol (Lu et al. 2025a) (Table 2). This finding underscores that generating tailored analytical standards is an essential step for accurately mapping and troubleshooting complex metabolic pathways (Lu et al. 2025a).

The accurate identification of a metabolic bottleneck is the critical first step, enabling a range of powerful strategies for its resolution. One solution is to screen for superior enzyme homologs from diverse species. For instance, selecting a highly active 7-dehydrocholesterol reductase (DHCR7) was instrumental in redirecting metabolic flux away from competing pathways (Qian et al. 2020) (Table 2). A more elegant solution is the discovery of novel enzymes that simplify the pathway. Researchers found that *Ajuga reptans* DWARF1 (ArDWF1) directly converts 24-methylenecholesterol to campesterol in a single step, bypassing the less efficient two-step reaction catalyzed by orthologs like OsDWF1 (Takemura et al. 2017). Beyond screening, introduced enzymes can be optimized through protein engineering. For example, in the effort to produce the brassinolide precursor 24-epi-ergosterol, the directed evolution of a plant-derived sterol reductase, DWF1, was a crucial step in establishing the novel biosynthetic pathway in yeast (Jiang et al. 2023) (Table 2).

### Managing sterol homeostasis and cellular toxicity

As phytosterol production increases, maintaining cellular health becomes paramount. The overaccumulation of free sterols is often toxic, prompting the cell to convert them into steryl esters. This process, mediated by acyltransferases (Are1p, Are2p), creates a metabolic sink that reduces the yield of free phytosterols. Consequently, a key strategy involves manipulating this homeostasis. Deleting *ARE1* and *ARE2* has been shown to augment the concentration of free sterols (Xu et al. 2023).

However, a complete lack of esterification can also be detrimental to cell growth and productivity. More sophisticated approaches now focus on fine-tuning this balance rather than simply blocking it. This can involve partially reinstating sterol acyltransferase activity to find an optimal state between production and viability (Xu et al. 2023) (Table 2). A landmark example of this advanced strategy was demonstrated in the production of 24-epi-ergosterol. Instead of deleting storage pathways, researchers precisely engineered sterol homeostasis by keeping the key acyltransferase ARE1 intact while overexpressing ARE2 and the steryl ester hydrolases YEH1 and YEH2. This created a dynamic "push-and-pull" system that actively managed sterol pools, preventing toxicity while maximizing flux towards the final product. This finely tuned strategy was instrumental in achieving an exceptional titer of 2.76 g/L in fed-batch fermentation and serves as a powerful model for the bulk production of other valuable phytosterols (Jiang et al. 2023) (Table 2). Building on this, the most successful campesterol production to date was achieved not by blocking storage, but by "restoring" sterol homeostasis. This strategy of optimizing the cell's capacity to handle excess sterols was a key factor in reaching a groundbreaking titer of 5.88 g/L in fed-batch fermentation (Liu et al. 2025) (Table 2), a result suggests that enabling the cell to safely store the product is superior to simply preventing storage.

### Systems-level engineering for high-titer production

Achieving benchmark production levels requires integrating all the above strategies at a systems level. A recent study by Qin et al. (2024) provides a masterclass in this approach. Their work began by selecting an optimal host strain with high lipid content and identifying the most effective promoters for key biosynthetic pathway genes. They then addressed intermediate-induced inhibition by modularly expressing sterol biosynthetic genes (ERGs), boosting the campesterol titer to 88.3 mg/L. Given that campesterol is a lipid-soluble molecule, the authors enhanced lipid droplet formation by introducing regulators for lipid droplet biosynthesis, thereby increasing campesterol production to 169.20 mg/L in shake-flask cultures (Qin et al. 2024) (Table 2). Finally, by optimizing metal cation regulation and transitioning to fed-batch fermentation in a 5 L bioreactor, they achieved an unprecedented titer of 1.44 g/L of campesterol (Qin et al. 2024). These integrated engineering efforts not only set a new benchmark for heterologous phytosterol production but also demonstrate that combining lipid droplet formation with metabolic engineering is an effective strategy for enhancing campesterol production and should apply to the production of other phytosterols and derivatives. We may adopt a more optimistic outlook regarding the industrial production of phytosterols. For other sterol-related products, such as vitamin E, the industry has already employed microbial fermentation to convert terpenoids into products at industrial scale (Ye et al. 2022).

### Advanced applications: from single molecules to functional blends

While research has traditionally concentrated on maximizing the yield of single phytosterol molecules, the transformative potential of this technology resides in the rational design and synthesis of customized, functional sterol blends engineered to address specific biological challenges. This approach redefines heterologous production, evolving it from a mere chemical manufacturing platform into a sophisticated tool for ecological engineering and bolstering food security.

A recent study by Moore et al. (2025) provides a compelling example of this approach. To address the widespread malnutrition in bees caused by pollen scarcity, the researchers first identified a critical nutritional gap: the absence of essential sterols required for brood-rearing in conventional artificial feeds. Moving beyond a single-sterol solution, they began by precisely analyzing the specific sterol profile vital for bee development. Subsequently, they engineered the yeast *Yarrowia lipolytica* to synthesize a customized blend designed to match this natural profile (Table 2). When this engineered yeast was incorporated into the bee diet, it was demonstrated to significantly extend brood production, thereby sustaining colony health even in the absence of natural pollen (Moore et al. 2025).

This work represents a profound shift in the goals of metabolic engineering. The focus is no longer on simply achieving a high titer of one compound, but on designing and producing a complex, multi-component product tailored for a specific biological function. This approach not only provides a technological solution to support critical pollinators but also opens up possibilities for creating other high-value nutritional supplements and functional ingredients and even mixtures of perfumes or volatile organic compounds emitted by certain flowers, marking a new era for microbial cell factories.

## Summary and prospect

Recent advancements in the study of phytosterol biosynthesis have propelled the field forward, spurred by the diverse applications of these compounds in sectors such as food supplementation, nutraceuticals, and pharmaceuticals. Phytosterols are critical for the synthesis of a myriad of steroidal compounds, with squalene recognized as an essential biosynthetic intermediate. Research efforts have concentrated on enhancing the accumulation of phytosterols using strategies that include gene manipulation, enzyme engineering, metabolic engineering, and the exploitation of various host organisms like bacteria, algae, and yeast. The prospects for phytosterol biosynthesis research are bright, with numerous opportunities for continued innovation and exploration.

**Fig. 4:**
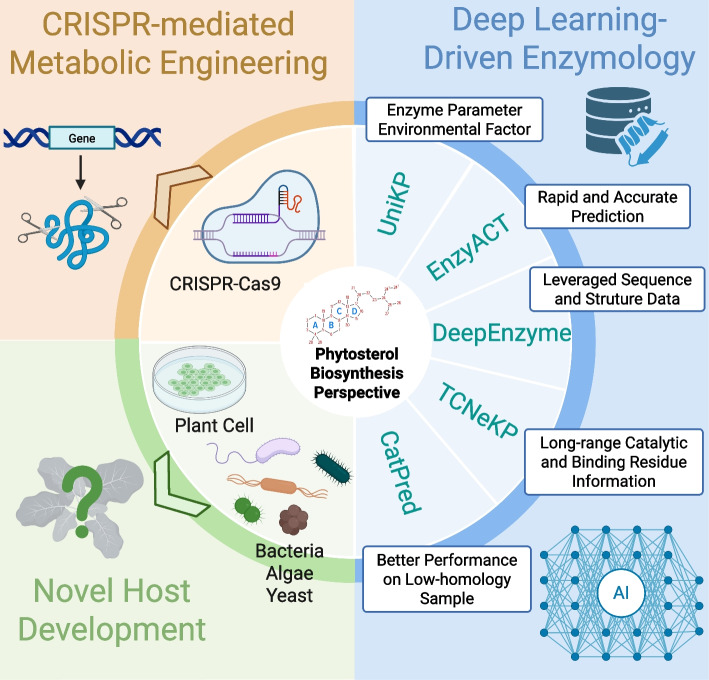
Prospects of phytosterol metabolic engineering by integrating CRISPR technology and deep learning models. This schematic depicts three key future directions for efficient phytosterol biosynthesis: (1) CRISPR-mediated precision metabolic engineering: Utilizing the CRISPR-Cas9 system to edit key genes in plant cells or other hosts to optimize metabolic pathways. (2) Novel host development: Transferring the phytosterol biosynthetic pathway into fast-growing and easily cultivable microorganisms (bacteria, yeast, algae) to create "cell factories". (3) Deep learning-driven enzymology: Applying various AI models for rapid and accurate prediction of enzyme sequences, structures, and functional parameters. The synergy of these three approaches aims to achieve sustainable and efficient production of phytosterols. Created in https://BioRender.com

A particularly promising avenue is the ongoing refinement of metabolic pathways via state-of-the-art synthetic biology methods. Techniques such as CRISPR/Cas9 and other gene-editing tools have been employed to meticulously adjust biosynthetic genes and optimize metabolic pathways (Fig. 4). Additionally, the pursuit of novel host organisms for phytosterol production is a burgeoning area of interest. While current hosts such as bacteria, algae, and yeast have been productive, the exploration of other microbial systems or plant cell cultures may unveil unique metabolic advantages conducive to enhanced phytosterol production. Identifying organisms with inherently high lipid content or particularly efficient sterol biosynthesis could further improve phytosterol yields (Fig. 4). Through the construction and analysis of metabolic networks and the simulation of various perturbations, researchers can uncover the intricate regulatory mechanisms that control phytosterol biosynthesis. These insights are expected to inform and steer the rational design of metabolic engineering strategies.

Looking ahead, it is anticipated that deep learning models will increasingly be employed to identify key enzyme active sites. The advancement of deep learning techniques has led to the development of various machine learning models designed to predict enzyme parameters and facilitate enzyme evolution (Fig. 4). One notable framework is UniKP, which utilizes pretrained language models to predict enzyme kinetic parameters while accounting for environmental factors such as pH and temperature during directed evolution tasks (Yu et al. 2023). Another significant approach is EnzyACT, which combines graph-based methodologies with language models to provide a rapid and accurate prediction of the effects of single or multiple mutations on enzyme activity (Li et al.2024b). While both UniKP and EnzyACT primarily utilize protein sequences as input, incorporating protein structural information could further enhance the accuracy of predictions. DeepEnzyme represents a pioneering advancement in this area, integrating the latest Transformer architecture with Graph Convolutional Networks (GCN) to effectively leverage both sequence data and the three-dimensional structural information of proteins (Wang et al. 2024c). Also, TCNeKP utilizes TCN for enzyme sequence feature extraction, effectively capturing long-range catalytic and binding residue information, which leads to superior performance on both wild-type and mutant enzymes, outpacing models like UniKP. However, its ability to model extremely long sequences is limited, potentially restricting its use for very large proteins (Lei et al. 2025). Meanwhile, CatPred excels in predicting enzyme kinetic parameters (kcat, Km, Ki) by comparing features from pre-trained protein language models, demonstrating strong performance on low-homology samples (Boorla and Maranas 2025). This advancement is expected to deepen our understanding of enzyme structure and function, thereby making substantial contributions to the field of enzyme engineering.

The field of phytosterol biosynthesis stands on the cusp of remarkable growth and innovation in the years to come. With unwavering research and development efforts, we anticipate the emergence of increasingly efficient production platforms and the proliferation of phytosterol applications across a broad spectrum of industries.

## Data Availability

All data generated or analyzed during this study are included in this published article.
